# Subjective Memory Evaluation before and after Temporal Lobe Epilepsy Surgery

**DOI:** 10.1371/journal.pone.0093382

**Published:** 2014-04-01

**Authors:** Chin-Wei Huang, Brent Hayman-Abello, Susan Hayman-Abello, Paul Derry, Richard S. McLachlan

**Affiliations:** 1 Department of Clinical Neurological Sciences, Western University, London, Ontario, Canada; 2 Department of Neurology, National Cheng Kung University Hospital, College of Medicine, National Cheng Kung University, Tainan, Taiwan; Utrecht University, Netherlands

## Abstract

Subjective memory (SM), a self-evaluation of memory, in contrast to objective memory (OM) measured by neuropsychological testing, is less well studied in patients with epilepsy. We assessed SM before and after temporal lobectomy. The Frequency of Forgetting 10 scale (FOF-10), developed to evaluate SM in dementia, was given before and one year after temporal lobectomy. Reliability and validity for use in epilepsy were first assessed. Measures of depression (CES-D) and neuroticism (PANAS) were done before and after surgery as well as complete neuropsychological assessment of OM. Correlation analysis between FOF-10 results and all the other variables was implemented. In 48 patients the FOF-10 was reliable and valid showing high internal consistency in all items (Cronbach's alpha >0.82) and high reproducibility (*p*<0.01). The FOF-10 also correlated with the memory assessment clinics self rating scale (MAC-S) (*p*<0.01). FOF-10 scores improved or were unchanged postoperatively in 28 patients (58%) and worsened in 20 (42%). The FOF-10 did not significantly correlate with memory scores from neuropsychological testing but did correlate with perceived word finding difficulty (p<0.001) and postoperative depression (p<0.05). A reduction in number of antiepileptic drugs (AEDs) after surgery distinguished those with improved postoperative SM. No correlation was found between SM and neuroticism, side of surgery or number of seizures. The FOF-10 is a brief and reliable measure of subjective memory in patients with epilepsy. Perceived memory impairment reflects more emotional state, language problems and quantity of AEDs than actual defects in memory function. These results would potentially be useful in presurgical counselling and management of memory issues after temporal lobe surgery.

## Introduction

Cognitive dysfunction, especially with respect to memory, can be a major complicating feature in epilepsy and can represent an important management challenge [1]. Memory problems have a multifactorial origin reflecting type of epilepsy, etiology, comorbid conditions and adverse effects of drug treatment [2].

Subjective memory (SM) concerns are important to the epilepsy population but are under recognized by treating physicians [3] and less extensively studied than objective memory (OM) as assessed by formal neuropsychological testing. In general, those with epilepsy are more likely to report memory problems than is the general population [4]. In the clinic, both the patient's perception as to whether they have a memory problem or not and the results of neuropsychological testing should determine changes in management. There are numerous tests which can be used to evaluate a person's OM performance but the number of measures designed to assess SM is still limited. The Memory Assessment Clinics Self rating Scale (MAC-S) is commonly used by clinicians to evaluate SM. It includes 21 ability-to-remember items, 24 items assessing frequency of occurrence of memory failures, and 4 global rating items assessing overall comparison to others, comparison to the best one's memory has ever been, speed of recall and concern or worry over memory function [5], [6]. The questionnaire is a broad measure of self-rated memory consisting of a large number of items, making it a relatively tedious task. In addition, this questionnaire includes several items sharing similarities.

The Frequency of Forgetting-10 scale (FOF-10), a 10-item Rasch modeled scale to measure memory self-efficacy, was developed from the 33-item frequency of forgetting scale of the Memory Functioning Questionnaire (MFQ) [7]. This shorter, more straightforward test is predicted by the same covariates as the long version, and has construct validity established in dementia. The questionnaire corresponds to real-life events, is easy to understand and can be self-administered in approximately 5-10 minutes. This measure has been mainly used in evaluating SM in people with Alzheimer's dementia [8], but has not been studied in patients with epilepsy. There are several studies of SM in epilepsy but those specifically focused on patients undergoing epilepsy surgery are few [9], [10]. Previous studies using different SM scales and some domains of OM evaluation (mainly verbal memory) inconsistently showed poor correlation between SM and OM [6], [9], [11]–[14]. Which domain of OM change could correlate with SM change after epilepsy surgery remains not completely known. Previous studies have suggested correlation between SM and mood, especially depression [15], [16].

This is the first study done on patients with epilepsy using the FOF-10. We first aimed to investigate the reliability of the FOF-10 by studying its test-retest stability and internal consistency, as well as to externally validate the instrument by coadministration of the MAC-S, a validated test of SM in patients with temporal lobe epilepsy (TLE). We then assessed the utility of FOF-10 in determining SM before and after surgical treatment. We also determined what factors might influence patient perception of their memory function. Three domains of measurements and investigations were completed before and one year after temporal lobectomy in patients with intractable TLE: (1) subjective memory; (2) comprehensive neuropsychological evaluation; (3) measures of depressive and neurotic symptoms.

## Methods

This study, including its consent procedure, was approved by the Western University ethics review board. A consent form was signed by all subjects and co-signed by a parent or guardian for those under age 18 years (only one subject was a minor). As indicated in the consent form, all data was anonymous with subjects identified only by an ID number. The signed and dated consent forms were retained by the investigator with copies being retained by the subject.

Subjects were recruited by an epileptologist from inpatients admitted to an 8-bed epilepsy monitoring unit at London Health Sciences Centre for investigation of surgical treatment of their epilepsy. The inclusion criteria were: at least 16 years of age; intelligence quotient higher than 70; a diagnosis of TLE and English as first language. Patients were excluded if they had a major psychiatric disorder, had previous surgery or if they were unable or unwilling to provide written informed consent.

### Outcome measures

The primary outcome measure was mean memory score of the FOF-10 before surgery (T1) and one year after surgery (T2), a higher score indicating better perceived memory. In addition, all patients underwent evaluations of depression and neuroticism as well as neuropsychological testing of OM. The first evaluation was done preoperatively and the second evaluation at one year postoperatively.

### Evaluation of Subjective Memory

After obtaining informed consent for participation in the study, the two questionnaires assessing SM (FOF-10 and MAC-S scale) were administered one to two months preoperatively. FOF-10 was re-administered within one week to assess test-retest reliability. FOF-10 at T2 (one year after surgery) was analyzed for all patients and stratified by right or left side surgery. Correlation analysis of T2 FOF-10 with clinical variables was performed.

Subgroup comparison was made of those who had higher T2 FOF-10 scores compared to T1 suggesting improvement (Group A) versus those with lower T2 FOF-10 scores suggesting worsening (Group B).

In addition to the standard FOF-10 memory questionnaire, we asked additional questions on general outcome after epilepsy surgery including perception of memory change, quality of life and overall surgery impact (see Addendum S1). Answers were assessed utilizing a Likert scale.

### Reliability and validity test of FOF-10

To test consistency and reliability of FOF-10 in patients with epilepsy, internal consistency and test-retest reliability were assayed. Internal consistency was determined and expressed as a Cronbach's alpha value [16], the most popular index of reliability for estimating the internal consistency of multiple items on a composite scale [17]. Test-retest reliability was determined using intraclass correlation coefficient (ICC) and Pearson Correlation coefficient. For validity, FOF-10 total score was compared respectively with MAC-S and correlation analysis was performed for consistency. The ability domain (items 3 and 6) in FOF-10 was correlated with the ability domain and four global items in the MAC-S scale. The frequency domain (items 4 and 5) were correlated with the frequency domain in MAC-S scale.

### Evaluation of seizure parameters

The main demographic variables (age, gender, education, handedness) of all the patients were evaluated and recorded. For each patient, the main characteristics of epilepsy (age of onset, side of epileptic focus, etiology, duration of epilepsy, number of seizures, number of AEDs, interictal and ictal findings in electroencephalography (EEG), brain magnetic resonance imaging (MRI) findings, surgical pathological findings) were recorded. Each of these variables was correlated with the FOF-10 score. To describe seizure outcome at T2, the seizure free rate was calculated.

### Surgical procedure

All the patients recruited in this study underwent a standard left or right temporal lobectomy including 2–3 cm of hippocampus.

### Evaluation of depression and neuroticism

Depression was evaluated using the Center for Epidemiological Studies Depression Scale (CES-D) [18]. The total score for CES-D is between zero and 60. A higher score suggests more depression, and clinical depression is deemed more likely if the score is above 16. Neuroticism was evaluated using the negative affect score from the Positive and Negative Affect Schedule (PANAS) [19]. The score consists of a number of words that describe different feelings and emotions. It ranges from 10–50, with lower scores representing lower levels of negative affect. Correlation analysis was then done to investigate if FOF-10 and CES-D or FOF-10 and PANAS were associated.

### Neuropsychological assessment

At both T1 and T2, patients underwent complete neuropsychological evaluation including several measures of memory and language: the Wechsler Abbreviated Scale of Intelligence (WASI; verbal intelligent quotient (IQ), performance IQ, full scale IQ), Wechsler Memory Scale – Third Edition (WMS-III; auditory immediate memory, visual immediate memory, auditory delayed memory, visual delayed memory), California Verbal Learning Test- 2nd Edition (CVLT-2) total learning (immediate memory), CVLT-2 long delay free recall, Rey-Osterrieth complex figure test delayed recall performance, Boston Naming Test, animal fluency and lexical fluency. Correlations were then calculated between FOF-10 scores and these objective neuropsychological results.

### Statistical analysis

SAS 9.1 was used for statistical analysis. Data were expressed as the mean ± standard deviation (SD). Statistical significant was deemed with the level of significance *p*<0.05. For correlation analysis, in addition to Pearson's Correlation coefficient, Kendal's Tau was used if the data did not show normal distribution. Spearman's rho was used for some analysis of non-parametric data.

## Results

A cohort of 48 patients out of an original 55 with TLE completed the questionnaires both preoperatively (T1) and one year after surgery (T2). Seven patients who did not complete the one year follow-up questionnaires were excluded. Mean age was 39 (16–63) years and 52% were female. Surgery was on the left in 27 and on the right in 21. After one year, 73% were seizure free or had auras only. Perceived quality of life was significantly better after surgery with 21 patients indicating improvement, 22 the same and 4 worse (p<0.01). Whereas 76% of subjects indicated they would have surgery again, 12% were hesitant to do so and 12% were not sure. Of those who would have surgery again 85% were seizure free after one year compared to 45% seizure free in those hesitant or not sure.

### Internal consistency, test-retest reliability and validity of the FOF-10

Of the 48 patients who did both the FOF-10 and MAC-S questionnaires 32 also completed the re-test of FOF-10 within one week. It was shown that the FOF-10 had a high internal consistency. The Cronbach's alpha value of total score index was 0.934. It also showed good reproducibility (ICC value, ranging from 0.671 to 0.935; Pearson correlation coefficient (r), ranging from 0.505 to 0.875, all *p*<0.005). An excellent consistency suggested this questionnaire was effective. The correlation coefficient between FOF-10 total score and MAC-S (ability, four global items and frequency) showed a general excellent consistency (*p* values ranging from <0.001 to 0.03). These results suggested that in general, FOF-10 had high reliability and validity in patients with TLE.

### Subjective memory analysis

**Figure 1:**
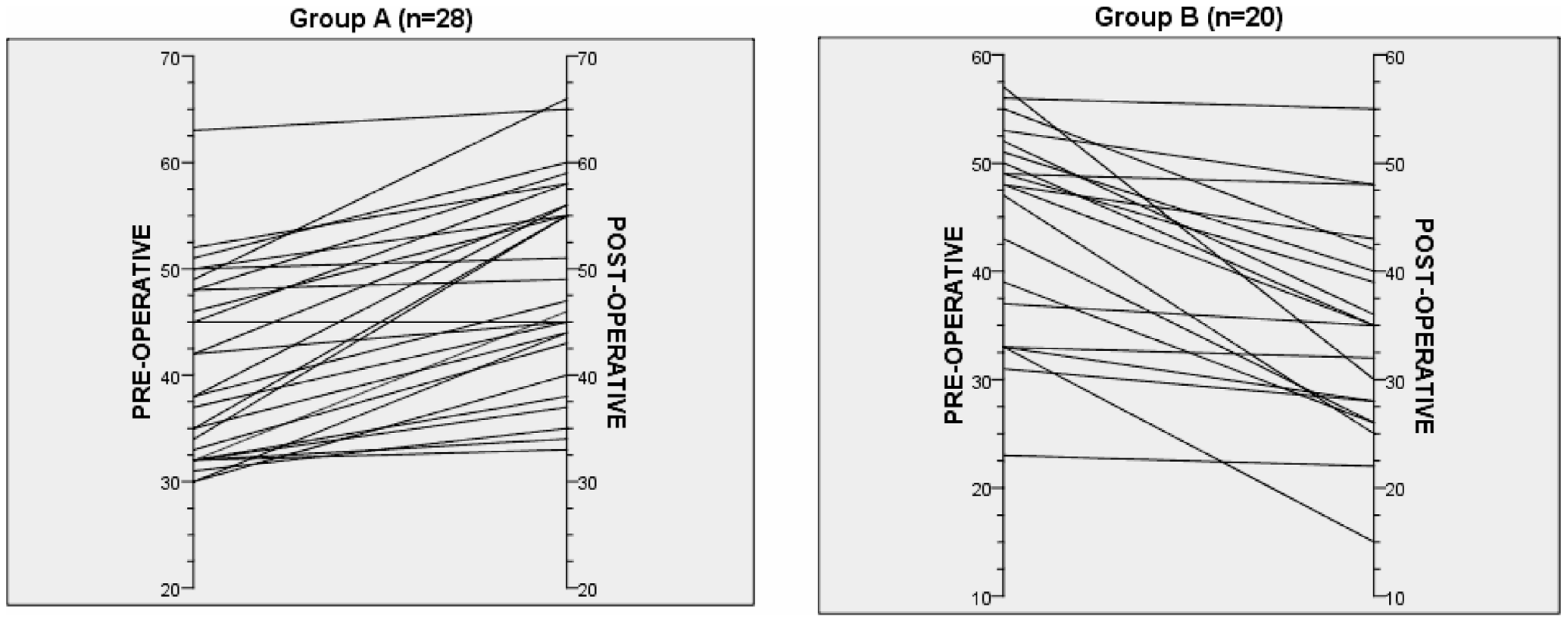
FOF-10 scores before and 1 year after surgery. Group A had improved or the same scores and Group B had worsening on follow-up.

Using FOF-10 to assess SM, scores ranged from 10 to 70 with lower scores indicating perceived poorer memory. The FOF-10 mean score for all patients was 42.9±9.0 preoperative and 44.2±12.3 one year after surgery reflecting no significant difference (*p* = 0.52). The postoperative FOF-10 was predicted by the preoperative FOF-10 (*r* = 0.439, *p*<0.01). Figure 1 shows that, compared to their preoperative FOF-10 scores, 28 patients (58%) had higher or the same (in 1 patient) FOF-10 scores postoperatively (Group A) and 20 (42%) had lower scores (Group B). FOF-10 scores correlated with qualitative assessment of SM as addressed in Question 8 to which 57% of subjects responded their memory was the same or better after surgery (FOF-10 48.9±9.7) while 43% thought it was worse (FOF-10 35.4±10.2; p<0.01). FOF-10 scores also had significant positive correlations with perceived word finding difficulty both before (Spearman's rho r = 0.43, p<0.01) and after surgery (Spearman's rho r = 0.72, p<0.001). There was no significant difference in FOF-10 scores between right and left TLE either before (*p* = 0.94) or after (*p* = 0.77) surgery.

Those who achieved better SM (Group A) versus those who had worsened SM (Group B) one year postoperatively (Table 1) had similar preoperative FOF-10 scores (40.43±8.70 vs 44.35±9.73), years of education (13.4±2.4 vs 12.8±2.8), age (39.3±12.5 vs 39.0±13.9), age of onset (16.0±13.7 vs 19.5±16.8), female gender (54% vs 50%), laterality (right in 46% vs 40%), right handedness (86% vs 90%), mesial temporal sclerosis on MRI (61% vs 70%), bi-temporal spikes in EEG (75% vs 80%), and subdural/depth electrodes insertion (25% versus 30%). In terms of seizure frequency, there was no significant difference between the two groups in simple partial seizures, complex partial seizures, generalized seizures or all seizures. Seizure outcome postoperatively was also the same in the two groups.

**Table 1:** Those with stable or improved SM (Group A) versus those with worsened SM (Group B) 1 year after surgery, in terms of seizure related factors, MAC-S and general SM questions.

	Group A (n = 28)	Group B (n = 20)	
	Mean (SD)	Mean (SD)	*p*
Fof10 difference (T2-T1)	8.64 (6.04)	−9.95 (7.77)	<0.001
T1 FOF-10	40.43 (8.70)	44.35 (9.73)	0.150
Education (years)	13.4 (2.4)	12.8 (2.8)	0.423
Age	39.3 (12.5)	39.0 (13.9)	0.948
Age of onset	16.0 (13.7)	19.5 (16.8)	0.441
Female (%)	54	50	0.807
Right TLE (%)	46	40	0.658
Handedness (Right) (%)	86	90	0.658
MTS on MRI (%)	61	70	0.507
MTS in surgical pathology (%)	50	50	1.000
Bi-temporal spikes in EEG (%)	75	80	0.684
History of depression (%)	50.00	31.25	0.348
Number of seizures per year at T1	54 (116)	78 (96)	0.531
Subdural electrodes inserted (%)	15	12.5	0.701

The number of AEDs used (Table 2) was significantly higher in Group A than in Group B preoperatively (2.3±0.79 vs 1.8±0.52; *p*<0.05) but not postoperatively (1.7±0.62 vs 1.5±0.62; *p* = 0.45). The number of patients using topiramate was similar in the two groups before and after surgery (*p*>0.1).

**Table 2:** AED use in Groups A and B.

	Group A (n = 28)	Group B (n = 20)	
	Mean (SD)	Mean (SD)	*p*
Number of AEDs at T1	2.3 (0.79)	1.8 (0.52)	0.033
More than 3 AEDs at T1 (%)	22.9	2.1	0.007
Number of AEDs at T2	1.7 (0.62)	1.5 (0.62)	0.451
Topiramate use at T1 (%)	18.4	7.9	0.275
Topiramate use at T2 (%)	12.8	7.7	0.697

In terms of depression, both groups had similar CES-D scores before (18.0±8.8 vs 16.2±8.3, *p* = 0.47) and after surgery (14.6±11.4 vs 17.0±12.0, *p* = 0.49). Of note, the FOF-10 score did not correlate with the CES-D score preoperatively (*r* = −0.06, *p* = 0.67) but did so after surgery (*r* = −0.28, *p* = 0.05). Similarly, both groups had similar PANAS scores for neuroticism before (21.6±6.6 vs 20.1±10.3, *p* = 0.55) and after surgery (17.2±7.5 vs 16.2±5.6, *p* = 0.62). The FOF-10 scores did not correlate with PANAS scores either at T1 (*r* = −0.190, *p* = 0.19) or T2 (*r* = −0.177, *p* = 0.24).

### Subjective versus objective memory assessment

Results of neuropsychological test performance for Group A and Group B, preoperatively and postoperatively, appear in Table 3. There was no significant correlation of T1 FOF-10 scores with objective neuropsychological evaluation at T1 (all *r* = −0.083 to 0.148, all *p*>0.05). Examining relationships between the SM change (T2 FOF-10 minus T1 FOF-10 score) with the OM change (T2 score minus T1 score in each domain) showed no significant correlations between the SM change and the OM change (*r* = −0.156–0.237, all *p*>0.05).

**Table 3:** Neuropsychological test performance for patients with stable or improved SM versus those with worsened SM 1 year postoperatively.

	Group A (n = 28)	Group B (n = 20)	
	Mean (SD)	Mean (SD)	*p*
**Objective memory at T1**			
Verbal IQ	98.61 (10.26)	93.3 (11.87)	0.105
Performance IQ	103.50 (14.15)	99.45 (13.82)	0.329
Full scale IQ	101.07 (11.26)	96.00 (12.59)	0.150
Auditory immediate memory	94.68 (13.53)	90.35 (13.32)	0.277
Visual immediate memory	97.03 (14.75)	95.50 (13.50)	0.714
Auditory delayed memory	94.11 (16.78)	89.05 (16.74)	0.312
Visual delayed memory	96.79 (16.05)	96.75 (14.98)	0.994
CVLT-2 total learning	50.32 (13.06)	43.00 (8.48)	0.049
CVLT-2 long delay free recall	−0.38 (1.44)	−1.32 (1.26)	0.035
ROCF test delay recall	−0.572 (0.90)	−1.174 (0.86)	0.030
Boston Naming Test	40.071 (10.69)	38.75 (12.17)	0.692
Animal fluency	41.39 (9.67)	35.53 (10.29)	0.065
Lexical fluency	41.04 (10.03)	40.06(9.51)	0.746
**Objective memory at T2**			
Verbal IQ	100.71 (11.89)	97.14 (14.01)	0.450
Performance IQ	107.56 (9.10)	106.43 (12.21)	0.767
Full scale IQ	103.67 (10.13)	101.86 (12.97)	0.660
Auditory immediate memory	97.00 (15.40)	89.21 (13.43)	0.149
Visual immediate memory	99.65 (16.74)	94.64 (15.12)	0.394
Auditory delayed memory	98.53 (16.45)	90.36 (12.94)	0.141
Visual delayed memory	97.29 (16.17)	96.21 (15.87)	0.853
CVLT-2 total learning	50.06 (15.06)	43.28 (10.31)	0.161
CVLT-2 long delay free recall	−0.413 (1.56)	−1.107 (1.33)	0.194
ROCF test delay recall	−0.802 (0.88)	−0.535 (0.91)	0.417
Boston naming test	41.29 (9.26)	40.71 (17.26)	0.208
Animal fluency	43.18 (11.73)	40.64 (10.03)	0.528
Lexical fluency	43.82 (8.76)	42.64 (7.07)	0.688

IQ: intelligence quotient; CVLT-2: California Verbal Learning Test-2nd Edition; ROCF: Rey-Osterrieth complex figure.

On objective neuropsychological testing, relatively fewer patients in Group A with perceived better memory after surgery had evidence preoperatively of bi-temporal dysfunction compared to those with decreased SM (Group B) but this did not reach statistical significance (*p* = 0.08). At preoperative testing, significantly lower OM performance on the CVLT-2 total learning (immediate recall), CVLT-2 long delay free recall, and ROCF delayed recall was seen in patients who then went on to have lower SM scores after surgery (Group B; Table 3). The groups did not significantly differ on any of the other cognitive measures preoperatively. Postoperatively, there were no significant differences between Group A and Group B on any of the neuropsychological test scores.

## Discussion

Our main findings in this study are summarized: 1) As an evaluation scale of subjective memory in patients with epilepsy, the FOF-10 was shown to have good reliability and validity; 2) Subjective memory as assessed by the FOF-10 did not correlate with neuropsychological measures of memory either before or after surgery; 3) Poorer SM was related to perceived word finding difficulty and a larger number of AEDs used preoperatively; 4) A correlation between SM and depression was found but only postoperatively and there was no correlation with degree of neuroticism.

Memory complaints are common in patients with epilepsy and sometimes can be of greater concern than the seizures [1]. Thus, a useful, easy-to-use and reliable measure to evaluate SM in epilepsy patients is desirable in clinical practice. This is the first study to use the FOF-10 to measure SM in patients with epilepsy. Patients with TLE were selected because they are more likely to express memory problems. The FOF-10 is constructed on a Rasch model which makes equating especially easy, though it may have a limitation with respect to guessing [20]. In this study, the Cronbach's alphas for all subscales were above 0.7, indicating good internal consistency for items in these subjective domains [21]. The stability of responses was estimated over a 1-week interval and similar score distributions on tests taken one week apart supported the stability of these subscales, which was verified by ICC and Pearson's correlation coefficient. In addition to the internal consistency and reliability, our validity analysis with MAC-S also established its general usefulness in patients with epilepsy.

Evidence for a correlation between SM and OM as measured by neuropsychological tests is limited [10]. It has been recently reported that SM deterioration in the absence of any reduced performance in OM tests is associated with altered hippocampal activity as measured by functional magnetic resonance imaging [22], suggesting SM impairment may reflect early neuronal dysfunction not measurable by OM testing. Alternatively, SM may reflect a longer time scale than is used for routine memory testing so that use of an extended retention interval may help to bridge the gap between SM and OM impairment [23].

Our results support other studies that showed no correlation between SM and OM (CVLT short and long delay free recall) [6], [9]. Although poor SM may sometimes be reported in patients with actual OM impairments, the inconsistent relationship between SM and OM suggests patients are misattributing other cognitive or emotional difficulties to memory problems. This has been shown in prior studies [12], [15] with, for example, SM being predicted by language performance, such as verbal fluency. Thus, patients may report problems with memory when in fact they have difficulty in generating words or interpreting their meanings. This was only partially seen in the current study; results did not show a significant relationship between SM and formal language measures, but SM and self-perceived word-finding difficulty were related.

Most previous reports have suggested subjective memory concerns relate to mood disorders particularly depression or to neuroticism both in the general population [24] and in epilepsy [4], [14], [25]–[27]. We did not find a significant correlation between SM and neuroticism but there was correlation with depression postoperatively. Neither pre-operative depression nor neuroticism predicted postoperative SM. In contrast to other measures of SM such as the MAC-S, the questions on the FOF-10 are more straightforward, addressing pure daily subjective memory complaints rather than more extensive issues that could relate to depression or neuroticism.

Earlier studies on SM in epilepsy showed generally no significant relationship between SM and clinical variables such as gender, chronologic age, epilepsy duration, seizure type, seizure frequency, and number of AEDs [14], [28]. However, there is a recognized tendency for patients on polytherapy to report greater cognitive difficulty, including perceived memory impairment, than do patients on monotherapy [29], [30]. Our study suggests that patients on polytherapy are more likely to note better SM outcome after epilepsy surgery when the number of medications has been reduced postoperatively. Although it has been noted that intractable epilepsy itself is often accompanied by SM decline [31], given the finding that both groups had similar seizure frequency and SM at baseline as well as seizure outcome after surgery, the number of AEDs rather than degree of seizure control would appear to be the more important contributing factor. We did not address the impact of specific drugs on SM with the exception of topiramate, an AED with a negative impact on cognition which is consistent with subjective complaints of patients [32]. Topiramate use did not reflect SM complaints in this study.

Laterality of the resected focus is a determinant of postoperative OM impairment [33] and possibly of self-awareness of SM [34]. Consistent with previous studies [10], [25], our data did not support a difference in SM performance between right and left TLE after surgery. One study suggested that TLE patients were more likely to show an improvement in their memory self-reports than to develop new complaints after surgery, regardless of laterality of lesion [6]. Our results are consistent with that finding since 28 subjects described better and 20 worse SM according to FOF-10 scores after surgery.

Considering recent emphasis on patient centered outcomes of clinical research, the results of this study will improve the dialogue between clinician's and psychologist's perception of memory impairment and that of patients. Clarification of the differences will potentially allow more effective measures to be utilized in alleviating the concerns of epilepsy patients and their families with respect to memory.

There is limitation in this study. Because of the limitation of the sample sizes which might lead to a reduced power, the group analyses were not done with methods like reliable change index. Given the fact the population of patients with surgically-indicated intractable temporal lobe epilepsy is relatively low, further prospective studies would be warranted.

In conclusion, subjective memory concerns should be taken into consideration when devising either medical or surgical treatments for seizures and appropriate measures taken to address what can be a major issue for many patients. However, clinicians should keep in mind that subjective memory complaints may reflect difficulties in domains other than memory per se (e.g., mood, language), and that the lack of complaints does not necessarily mean no memory impairment. The FOF-10 could serve as a reliable and useful tool for evaluation of SM in patients with epilepsy.

## Supporting Information

Addendum S1 **The Frequency of Forgetting 10 scale (FOF-10) questionnaire.**
(DOCX)
