# Supplementary material for: Subjective Memory Evaluation before and after Temporal Lobe Epilepsy Surgery
Source: PLoS One. 2014 Apr 1;9(4):e93382. doi: 10.1371/journal.pone.0093382 (PMC3972133; doi:10.1371/journal.pone.0093382)
Supplement: Addendum S1 — The Frequency of Forgetting 10 scale (FOF-10) questionnaire. (DOCX) [file pone.0093382.s001.docx]

**Addendum S1**

**QUESTIONNAIRE (Please circle your answer)**

1. **During the past 4 weeks, how much have you been bothered by memory difficulties?**

Not at all A Little Somewhat A Lot Extremely

1. **How has the quality of your life been during the past 4 weeks? That is, how have things been going for you?**

Very Well (could hardly be better) Pretty Good Good & Bad (about equal) Pretty Bad Very Bad (could hardly be worse)

3. ***How would you rate your memory in terms of the kinds of problems that you have?**

Major Problems Some Minor Problems No Problems

1 2 3 4 5 6 7

4. **How often do these present a problem for you?**

Always Sometimes Never

*Remembering names 1 2 3 4 5 6 7

*Remembering faces 1 2 3 4 5 6 7

*Remembering where you put something

1 2 3 4 5 6 7

*Remembering directions to places

1 2 3 4 5 6 7

*Beginning to do something and forgetting what you were doing

1 2 3 4 5 6 7

*Word-finding (words on the tip-of-my-tongue experience)

1 2 3 4 5 6 7

5. **As you are reading a novel, how often do you have trouble remembering what you have read…**

Always Sometimes Never

*The paragraph just before the one you are currently reading.

1 2 3 4 5 6 7

*The sentence before the one you are currently reading.

1 2 3 4 5 6 7

6. **How well do you remember things, which occurred…**

Very Bad Fair Very Good

*Between one and five years ago:

1 2 3 4 5 6 7

*Between six and ten years ago:

1 2 3 4 5 6 7

7. **In the past year:**

I have had no seizures or auras Yes No

I have had only auras Yes No

I have had seizures Yes No

If yes (to above question) how many _______________

*FOF-10 items marked with asterisks
